# Identifying the need for specialized palliative care in adult cancer patients – development and validation of a screening procedure based on proxy assessment by physicians and filter questions

**DOI:** 10.1186/s12885-019-5809-8

**Published:** 2019-07-01

**Authors:** Christoph Ostgathe, Kim N. Wendt, Maria Heckel, Sandra Kurkowski, Carsten Klein, Stefan W. Krause, Florian S. Fuchs, Christian M. Bayer, Stephanie Stiel

**Affiliations:** 1 Comprehensive Cancer Center CCC Erlangen – EMN, Friedrich-Alexander-Universität Erlangen-Nürnberg (FAU), University Hospital Erlangen, Erlangen, Germany; 2 Department of Palliative Medicine, Friedrich-Alexander-Universität Erlangen-Nürnberg (FAU), University Hospital Erlangen, Erlangen, Germany; 3 Department of Internal Medicine 5, Haematology and Oncology, Friedrich-Alexander-Universität Erlangen-Nürnberg (FAU), University Hospital Erlangen, Erlangen, Germany; 4 Division of Respiratory Medicine, Department of Internal Medicine 1, Friedrich-Alexander-Universität Erlangen-Nürnberg (FAU), University Hospital Erlangen , Erlangen, Germany; 5 Department of Gynecology and Obstetrics, Friedrich-Alexander-Universität Erlangen-Nürnberg (FAU), University Hospital Erlangen, University Breast Center Franconia, Erlangen, Germany; 6 Institute for General Practice, Hannover Medical School, Hannover, Germany

**Keywords:** Delivery of health care, Needs assessment, Palliative care, Cancer, Psychometric properties

## Abstract

**Background:**

One challenge in caring for cancer patients with incurable disease is the adequate identification of those in need for specialized palliative care (SPC). The study’s aim was to validate an easy to use phenomenological screening tool.

**Methods:**

The German tool is based on the National Comprehensive Cancer Network (NCCN) Palliative Care guidelines and contains ten items in five domains that focus e.g. on diagnosis, functional status, complications, comorbidities, and palliative care relevant problems such as symptom management, distress, and support of family and team members. Sum score ranges from 0 to 14 (no need to great need). Assessment to identify SPC needs was done in university hospital wards between 1 and 08/2017 by health care professionals on admission of the patient if the disease was incurable and expected prognosis < 12 months. The Integrated Palliative Outcome Scale (IPOS, staff version), an outcome assessment instrument for palliative care that consists of ten items, served as external criterion; in sub samples inter-rater/test-retest were performed.

**Results:**

Data from 208 patients with incurable disease and life expectancy < 12 months (54.8% female; average age 63.5 years, range 21–96) were assessed using the tool. The tool has good convergent validity; the correlation between the sum scores of IPOS and our tool showed a significant and substantial effect. The sum score was independent of the patient’s age, gender and primary diagnosis. Patients who already were in contact with SPC had significantly higher screening scores than patients without. With a cut point of  ≥ 5, 80.8% of the screened patients were in need for SPC. Cronbach’s alpha was α = .600. Rater agreement (inter-rater, test-retest) varied between single items. Correlation coefficients showed significant substantial effects.

**Conclusions:**

This is the first validation of a screening procedure in German language identifying SPC needs of adult patients with advanced cancer and the first using filter questions as a pre-screening. Proxy assessment of SPC needs by physicians in cancer care settings is feasible and the suggested tool presents a valid instrument to trigger a PC consultation.

**Trial registration:**

The study was not registered.

## Background

Comprehensive oncological treatment achieved ground-breaking advancement over the last decades and in many patients diagnosed with cancer survival has increased significantly. However, in a substantial number of patients cancer progresses and a need for specialized palliative care may evolve at some point during the disease trajectory. The Palliative Care (PC) approach offers – on a generalized or specialized level - continuity of care including symptom management as well as psychosocial and spiritual support for all patients with life limiting diseases [1]. Patients may be treated in parallel with oncological experts or - in case of more complex needs - exclusively by multi-professional specialized palliative care (SPC) professionals.

Here, one of the major challenges is the adequate identification of patients in need for SPC. Timely SPC integration has shown to improve quality of care, reduce costs, and may even increase patients’ survival time [2–4]. The causes for late referrals are multifaceted. Still oncologists and their patients may have an inadequate awareness of palliative care. Difficulties in prognostication may also hamper timely access. A recent follow-up study on patients cared for by a German inpatient SPC service at a tertiary center showed that less than 5% were seen for the first time more than 6 months before death and median survival from this first contact was 24 days [5]. To foster a fair and appropriate access for patients’ systematic screening on needs for SPC may be beneficial. Hence for Germany there is a scarcity of validated screening tools and that is one more cause that patients are often identified too late in the disease trajectory.

In cancer, the actual stage of the disease was proposed as a threshold to integrate SPC [6]. However, not every patient with advanced disease requires SPC and at the contrary, patients at an earlier stage may already have needs for SPC. For a mixed population with cancer and non-cancer patients general indicators for deterioration in health status and approaching death were developed and evaluated [7]. These indicators may help to determine the need for SPC and may prompt clinicians to initiate needs assessment and advance care planning. However, it may fail in identification of the need for SPC and in particular in daily routine.

The evidence on screening for the detection of the need to integrate SPC is scarce. For cancer patients, three different possible determinants are discussed:

First, the need for SPC could be defined by the actual disease/stage of the disease as proposed by Gaertner et al. [6], but this disease specific determination has not yet been further evaluated.

Second, the need for SPC could be defined by professionals. For that purpose, Glare/Chow [8] developed a phenomenological tool for professionals that classified 34% of all cancer patients in an outpatient clinic in need for SPC. Trout et al. [9] developed a rather medically oriented proxy tool combining primary disease, number of co-morbidities, the level of performance status defined by the Eastern Cooperative Oncology Group (ECOG) [10], and intensity of a set of core symptoms (pain, depression, fatigue, nausea, cognitive impairment, and dyspnoea). According to a - from our perspective artificially set - threshold, 13% of inpatients with cancer diagnosis were in need for a SPC consultation [9].

Thirdly, patients’ self-assessment could be used to identify the need for SPC. Morita et al. used patients’ self-assessment including the distress thermometer and identified 23% of cancer patients in need for SPC [11]. For a mixed population with cancer and non-cancer diagnosis, Highet et al. proposed prognosis as well as disease and non-disease specific determinants (e.g. weight loss, unplanned hospital admissions) to identify deterioration in health status and approaching death resulting in a need for PC in general [7]. In case of advanced organ failure they identified 41% of renal patients and 83% of patients treated by the liver unit to be in a deteriorated health status and approaching death [7]. All-in-all the level of evidence is low and the results of the existing studies are rather heterogeneous.

None of the instruments above are validated and routinely used for screening for SPC in Germany.

### Study aim

The aim of this project is to develop and validate an easy to use, routine and patient-centered strategy for identification of cancer patients in need for SPC taking disease and prognosis as well as the patients’, families’ and their carers’ needs into account. A practical and feasible screening instrument should be usable as proxy assessment with little workload burden for the clinicians as not all patients are capable of self-assessment. The manuscript presented here aims to improve clinical practice to trigger a PC consultation and foster the international discussion on screening for SPC.

## Methods

### Development of the screening tool

A scoping review of the literature on screening tools for SPC needs was conducted. The mode of development, mode of assessment, target groups, settings, identification criteria, and threshold/trigger points were collected. Based on these data, a patient-centered screening tool following the National Comprehensive Cancer Network (NCCN) criteria as described by Glare/Chow [8] with an implementation strategy was developed and discussed in a multidisciplinary (physicians, psychologist, nurse) and interdisciplinary (respiratory medicine, haematology/oncology, gynaecology and palliative medicine) expert group within our comprehensive cancer center. We decided on those disciplines following the competencies needed to successfully conduct the study: for the psychometric aspects we choose a psychologist, for the patient care and organizational aspects on conducting the screening we choose physicians and a study nurse. The four disciplines are part of the comprehensive cancer center and committed to participation in this project. The screening tool items used by Glare/Chow [8] were translated in German language by two German native speakers with high level of fluency in English independently. The two versions were compared and the final wording and examples were determined by the expert group. In order to avoid that all patients admitted to the participating units had to be fully screened, two filter questions were installed. Following the German “Evidenced-based Guideline: Palliative care for patients with incurable cancer” stating that “all patients must be offered palliative care following the diagnosis of incurable cancer, regardless of whether cancer-specific therapy is being implemented” screening should only be done for patients with incurable cancer [12]. As second filter the surprise question (with a cut off of 12 months life expectancy) was used [13]. The proposed phenomenological screening procedure contains the two filter questions followed by a German version of Glare’s working group tool [8, 14] with five domains including ten items (see Table 1).

**Table 1:** Screening tool

Items		Possible points	Points patient
Diagnosis: metastatic or locally advanced cancer		2	
Functional status score: ECOG^a^		0–4	
Fully active, able to carry on all pre-disease performance without restriction	0		
Restricted in physically strenuous activity but ambulatory and able to carry out work of a light or sedentary nature	1		
Ambulatory and capable of all selfcare but unable to carry out any work activities; up and about more than 50% of waking hours	2		
Capable of only limited selfcare; confined to bed or chair more than 50% of waking hours	3		
Completely disabled; cannot carry on any selfcare; totally confined to bed or chair	4		
one or more serious complications of advanced cancer usually associated with a prognosis of < 12 months (e.g. brain metastases, hypercalcemia, delirium, spinal cord compression, cachexia)		1	
one or more serious comorbid diseases also associated with poor prognosis of < 12 months (e.g. moderate-severe COPD or CHF, dementia, AIDS, end stage renal failure, end stage liver cirrhosis)		1	
Palliative care problems:		1 each	
Symptoms uncontrolled by standard approaches			
Moderate to severe distress in patient or family, related to diagnosis or therapy (personal purposes/expectations, educative or informational needs, cultural factors affecting the treatment)			
Patient/family concerns about course of disease and decision making (including realisation of power of attorney/patient decree)			
Patient/family requests palliative care consultation team			
Team needs assistance with complex decision making or determining goals of care (e.g. value/risks of treatment, desire to die)			
Prolonged length of stay (> average length of stay)			
Total score		0–14	

^a^ *ECOG* Eastern Cooperative Oncology Group Performance Status

### Study design and material

Ethical approval of the local ethics committee was obtained before starting the study. According to the data protection officer of the university hospital patient consent was not necessary. Cooperating wards had given written informed consent to the procedure of data collection. Between January and August 2017 the screening tool was applied in four cancer care settings of a university hospital: respiratory medicine, haematology/oncology, gynaecology and palliative medicine. All admissions to these settings were first assessed by a study nurse at the day of admission or the following working day if they met the inclusion criteria: i) aged ≥18 years, ii) treated in case of a cancer disease, iii) not assessed within this same trial during the last 3 months.

If evaluated positive for study inclusion, further assessment was triggered and the study nurse provided the ward with the necessary documents. One of the physicians caring for the patient at the study site answered the filter questions within three working days: i) incurable advanced cancer (yes/no), and ii) whether he/she would be surprised if the patient died within 1 year (surprise question) (yes/no).

In case the cancer disease was incurable (yes) and the surprise question was answered with ‘no’, the physician filled out the screening tool identifying SPC needs and the Integrated Palliative Outcome Scale for professionals (IPOS, staff version). IPOS is an instrument capturing the most important concerns of patients in PC e.g. symptom burden and psychosocial aspects. These aspects are rated from 0 (no effect) to 4 (overwhelmingly) and summarized into a sum score in which higher scores indicate lower outcomes [15]. For this study it was used as an external criterion as there are currently no standard measures that could be used to define the needs of SPC.

Subsamples at each site were employed to perform inter-rater (by two different professionals at the same assessment day) and test-retest (second rating by same professional within three to five working days after initial assessment) reliability for the screening tool. Subsample size depended on the return rate and 54 pairs were considered sufficient to significantly prove moderate agreement [16]. For test-retest subsample the ‘Self-care Index’ (Selbstpflegeindex, SPI) as an indicator of status deterioration was captured. SPI is part of the German routine assessment instrument “Outcome-oriented Nursing Assessment” (‘ergebnisorientiertes Pflege Assessment©’, ePA©) and is meant to predict the risk of insufficient care after discharge from hospital. The SPI consists of ten items and its sum score ranges from 10 (= maximum impairment of self-care) and 40 (= full ability of self-care) [17].

Additionally sociodemographic data such as age, gender, performance status (Eastern Cooperative Oncology Group (ECOG)) [10], primary diagnosis and connection to SPC at home or during hospital stay as well as decease during the survey period were collected. Previous or existing contact to SPC was registered and is to be regarded as an indication of existing SPC needs as those are not easily to determine otherwise.

To be 95% confident that problems with a probability of 10% will be detected [18] the target sample size for validation was calculated with 30–40 for each setting (∑90–120).

All physicians were instructed in using the screening tool before data collection. In order to support the implementation of such a screening tool, all recruiting sites were supported during the assessment phase by regular site visits and phone calls of the study nurse. Each time the study nurse brought new screening tools for admitted patients that fulfilled the inclusions criteria she checked if the other tools were conducted in time and reminded the physicians if necessary. Minimum frequency of visits was two per week. With every patient included in the study a contact on site to deliver the study documents and one more to collect the results took place.

### Data analyses

The instrument was tested in terms of psychometric validation with analysis of its validity and reliability in accordance with the recommendations of Streiner and Norman [19] regarding the main components of health measurement instrument validations. IBM SPSS Statistics 21 (SPSS Inc., Chicago, IL, USA) [20] for Windows was used for statistical analyses.

Results were considered significant if *p* < 0.05.

#### Validity

*Content validity* ensures that there are no important aspects of the targeted outcome missed by the scales [19]. In this study it arises out of the development of the screening tool.

For *construct validity convergent* and *discriminant validity* was evaluated. *Convergent validity* was checked by using Pearson correlations between screening score and sum score of IPOS as an external criterion based on the hypothesis that an instrument measuring comparable aspects should show a substantial effect with correlation values > 0.5 [21]. For *discriminant validity*, association between the screening tool and different patient- and disease-related aspects were examined with group comparisons. This was done to evaluate if the screening tool was sensitive to these patient- and disease-related factors. Differences in sum score of the screening tool between groups of patients with varying age (in groups), gender and primary disease were investigated. T-tests and one-way analysis of variance (ANOVA) were used.

*Criterion validity* was checked by using *concurrent and predictive validity.* For *concurrent validity* we analyzed whether the patient was already in contact to PC at home or during hospital stay as a criterion for group comparison expecting that admissions with connection to PC show higher screening scores. *Predictive validity* was evaluated by group comparison between admissions that deceased during the survey period and those who didn’t, using the hypothesis that deceased admissions present higher screening scores, due to a higher symptom burden close to death [22].

#### Reliability

For *internal consistency* Cronbach’s alpha and ‘alpha if item deleted’ were calculated for single screening items. Values between 0.7 and 0.9 were considered to indicate good psychometric values [23].

For *inter-rater* and *test-retest reliability* Cohens Kappa was used for single screening items. To guarantee a stable condition for test-retest only patients were included that showed differences in SPI ≤ 10% between first to second estimation. Values between 0.4 and 0.75 indicate good agreement [24]. For screening score Pearson correlations were used with values higher than 0.5 indicating a substantial effect [21].

#### Determining a cut point for SPC needs

We analyzed positive predictive values (PPV) and negative predictive values (NPV) by defining patients as in need of SPC by i) existing contact to PC unit, inpatient or outpatient consultation team or ii) ≥ 1 item was scored with 3 or 4, which is meant to require high clinical attention in the IPOS assessment [25]. We used a screening score ≥ 5 following Glare/Chow [8] and compared the results to higher and lower scores.

## Results

### Recruitment and study population

**Fig. 1:**
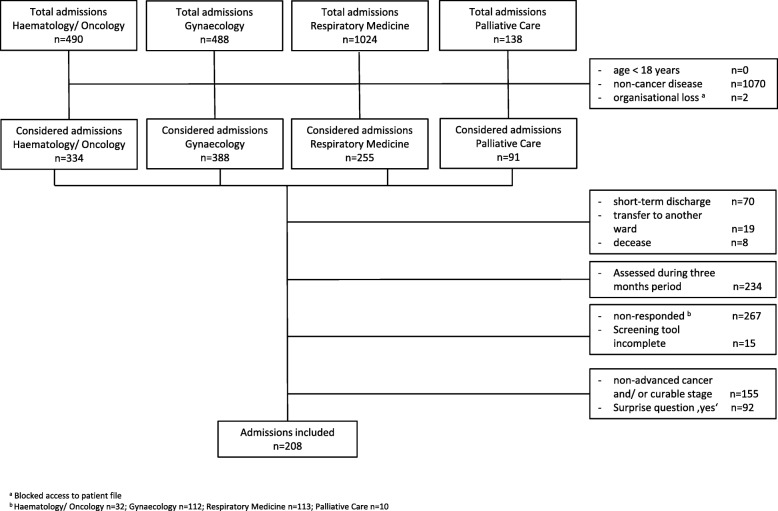
Recruitment. Legend: a Disabled access to patient file. b Haematology/Oncology *n* = 32; Gynaecology *n* = 112; Respiratory Medicine *n* = 113; Palliative Care *n* = 10

Between January 9th and August 31st 2017 a total of *n* = 2140 admissions at the 4 units were recorded. Considering the inclusion criteria, *n* = 1070 patients were excluded because of non-cancer-diseases and *n* = 2 because of organizational loss. Due to short-term hospital discharge (*n* = 70), transfer to another ward (*n* = 19) or rapid death (*n* = 8) *n* = 97 admissions dropped out. *N* = 234 admissions were excluded because they had already been assessed within this same trial during the preceding 3 months. For *n* = 737 admissions the study assessment was triggered and for *n* = 455 completed by the physician within three working days after admission. Further *n* = 247 patients were excluded after the two initial questions, because their cancer disease was non-advanced or curable (*n* = 155) or the surprise question was answered with ‘yes’ (*n* = 92). Remaining *N* = 208 patients were included for the validation process of the screening tool (see Fig. 1). *N* = 9 of these were screened twice because of multiple admissions with a greater interval than 3 months during the survey period. For *n* = 100 patients an estimation of two different professionals at the day of the initial assessment and for *n* = 76 patients an test-retest estimation of the same professional was performed.

The 208 patients were mainly female (54.8%) and mean 63.47 years (SD ± 13.383, range 21–96 years). Their performance status was mainly ECOG 2 (23.6%), ECOG 3 (19.7%) and ECOG 4 (20.7%) (see Table 2).

**Table 2:** Patient demographic and disease-related data and association with screening score

*N* = 208			Mean score ± SD	Test/Test statistic	Significance
Gender	male	45.2%	7.47 ± 2.413	t-Test: t_206_ = 1.615	*p* = .108
	female	54.8%	6.89 ± 2.722		*p* = .122
Age	Years; mean 63,47 ± 13,383 range 21–96	21–30	7.50 ± 2.121	one-way ANOVA (groups): F(7, 200) = 1.654	
		31–40	7.40 ± 1.776		
		41–50	8.47 ± 2.452		
		51–60	6.90 ± 2.564		
		61–70	6.91 ± 2.914		
		71–80	6.72 ± 2.447		
		81–90	8.25 ± 2.236		
		> 90	9.50 ± 0.707		
Performance status	ECOG 0	5.3%	3.09 ± 1.044	one-way ANOVA: F(4, 203) = 120.081	*p* < 0.01
	ECOG 1	30.8%	5.02 ± 1.386		
	ECOG 2	23.6%	6.78 ± 1.571		
	ECOG 3	19.7%	8.93 ± 1.233		
	ECOG 4	20.7%	10.09 ± 1.571		
Primary diagnosis	Bronchial carcinoma	16.3%	7.00 ± 2.807	one-way ANOVA: F(22, 185) = 1.405	*p* = .166
	Mammary carcinoma	14.4%	6.67 ± 2.758		
	Carcinoma of the female genital tract	9.1%	5.79 ± 2.149		
	Renal cell carcinoma	5.3%	7.36 ± 3.042		
	Myelomatosis	4.8%	5.70 ± 2.163		
	Leukemia	4.8%	6.30 ± 1.947		
	Colon cancer	4.3%	8.33 ± 2.179		
	Lymphoma	4.3%	6.56 ± 2.242		
	Esophageal cancer	4.3%	8.11 ± 1.453		
	Pancreatic carcinoma	4.3%	7.67 ± 2.598		
	others	28.1%	7.97 ± 2.575		
Connection to PC	yes	44.7%	9.27 ± 1.695	t-Test: t_201_ = −15.303	*p* < 0.01
	no	52.9%	5.44 ± 1.845		
	missing data	2.4%			
Decease during survey	yes	30.3%	8.59 ± 2.380	t-Test: t_206_ = −5.643	*p* < 0.01
	no	69.7%	6.52 ± 2.441		

### Screening score

**Fig. 2:**
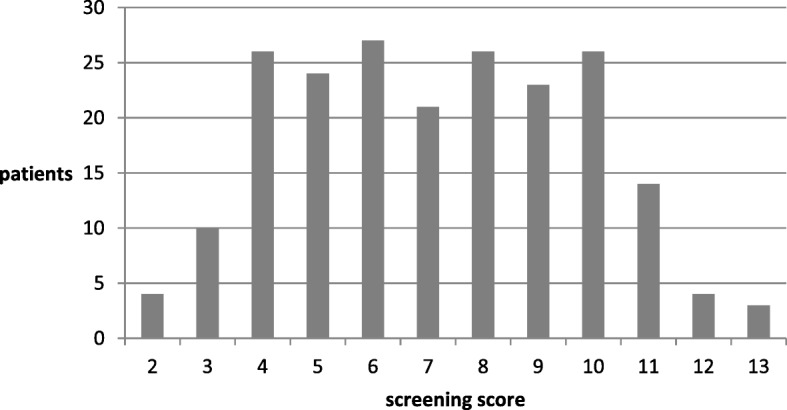
Screening score by patient

**Fig. 3:**
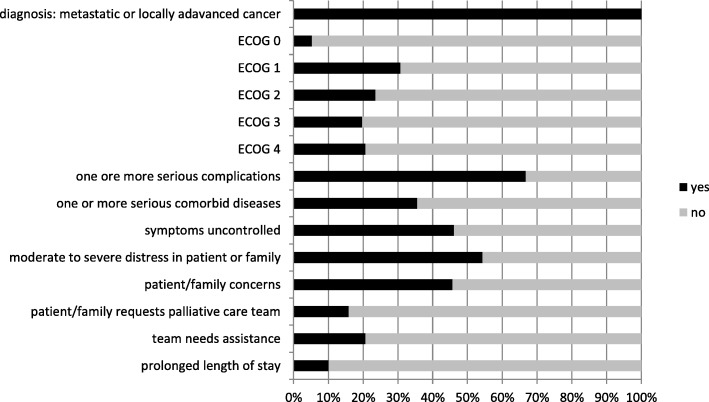
Answers per item. Legend: a ECOG = Eastern Cooperative Oncology Group Performance Status

The mean screening score was 7.15 (SD ± 2.597) with scores ranging from 2 to 13 (see Figs. 2 and 3).

### Psychometric properties

#### Validity

*Content validity* was ensured via a scoping review of the literature on screening tools for SPC needs and the systematic collection of trigger points content validity.

*Convergent validity* was tested with Pearson correlations between screening scores and IPOS sum score. To calculate the IPOS sum score < 50% of missing items were tolerated and imputed by a mean score based on the items completed. In *n* = 57 cases (27.4%) more than 50% of the IPOS items were missing and no sum score was calculated. The Pearson correlation coefficient shows a two-sided significant (*p* < 0.01) and substantial correlation effect (*r* = 0.547; *n* = 151).

For *discriminant validity* differences in screening score in patient- and disease-related aspects were evaluated. The physician’s estimation of the screening tool is independent of patient’s age (in groups) (F(7, 200) = 1.654, *p* = .122), gender (t_206_ = 1.615, *p* = .108) and primary diagnosis (F(22, 185) = 1.405, *p* = .166) (see Table 2).

*Criterion validity:* For *concurrent validity* screening scores of patients with connection to SPC by PC unit, inpatient or outpatient PC consultation team (*n* = 93; 44.7%, mean screening score 9.27 ± 1.695) and without connection to PC (*n* = 110; 52.9%, mean screening score 5.44 ± 1.845) were compared. *N* = 5 patients (2.4%) were excluded, because information about connection to PC at home were not available. Screening score is significantly higher of admissions with connection to PC (t_201_ = − 15.303, *p* < 0.01) (see Table 2). For *predictive validity* screening scores of patients who deceased during the survey period (*n* = 63, 30.3%, mean screening score 8.59 ± 2.380) and those who didn’t (*n* = 145, 69.7%, mean screening score 6.52 ± 2.441) were compared. Screening score of deceased patients shows a significant higher value (t_206_ = − 5.643, *p* < 0.01) (see Table 2).

#### Reliability

*Internal consistency* was calculated for the single items of the screening tool. Cronbach’s alpha was α = .600 and Cronbach’s alpha if item deleted ranged from .513 to .607 for each single item. Deleting the item ‘diagnosis: advanced or metastatic cancer’ would improve the internal consistency to .607.

*Inter-rater reliability* was tested for *n* = 100 patients. Cohens Kappa ranged from κ = .220 (patients’/ families’ concerns) to κ = .620 (patient/family asks for PC consultation). Pearson’s correlation coefficient shows a two-sided significant (*p* < 0.01) substantial effect (*r* = .745) (see Table 3).

**Table 3:** Cohen’s Kappa and correlation coefficient of inter-rater and test-retest analysis on single item level

Factors	Items	Inter-Rater (*n* = 100)		Test-Retest (*n* = 66)	
		κ	*p*	κ	*p*
Diagnosis	Diagnosis	^a^	^a^	^a^	^a^
ECOG	ECOG	.499	< 0.01	.535	< 0.01
Complications	Complications	.345	0.001	.318	0.007
Comorbidities	Comorbidities	.561	< 0.01	.402	0.001
Palliative relevant problems	Symptoms	.418	< 0.01	.545	< 0.01
	Patient/family distress	.540	< 0.01	.449	< 0.01
	Patient/family concerns	.220	.023	.262	0.030
	Patient/family requests PC consult	.620	< 0.01	.570	< 0.01
	Team assistance needed	.357	< 0.01	.041	0.735
	Prolonged hospital stay	.226	0.021	.168	0.138
		*r*	*p*	*r*	*p*
Screening Score		.754	< 0.01	.703	< 0.01

^a^ No calculation due to constant value

*Test-retest* reliability was performed for *n* = 76 patients. *N* = 10 patients were excluded due to a change of SPI values of more than 10% (*n* = 7) or because the SPI was not recorded (*n* = 3). Cohens Kappa ranged from κ = .041 (team assistance needed) to κ = .570 (patient/family request PC consultation) (see Table 3). Pearson’s correlation coefficient shows a two-sided significant (*p* < 0.01) substantial effect (*r* = .694).

### Determining a cut point for SPC needs

A screening score ≥ 5 showed a PPV of 56.7% referring to the need for SPC treatment evident due to a preexistent contact to PC unit, inpatient or outpatient consultation team. Hence, more than half of the patients with a score ≥ 5 were already in contact with SPC, and a NPV of 100%, implied that no patient would be missed.

Referring to IPOS estimations a screening score ≥ 5 had a PPV of 95.9% including that almost all patients with a score ≥ 5 had one or more symptoms that requires clinical attention. Nevertheless it had a NPV of 37.0% meaning that almost two-third of patients with ≥1 symptom that requires clinical attention would be missed.

Analyzing PPV and NPV for a cut point ≥4 in comparison, lead to lower values in total. Using a cut point ≥6 shows higher PPVs, but lower NPVs (see Table 4).

**Table 4:** Positive and negative predictive values (PPV and NPV) for different cut points

	Existing contact to PC		IPOS ≥ 1 item 3 or 4	
	PPV	NPV	PPV	NPV
Cut point 4	49.2%	100%	91.0%	29.1%
Cut point 5	56.7%	100%	95.9%	37.0%
Cut point 6	63.4%	95.1%	96.1%	22.9%

Using a score ≥ 5, in 80.8% of the preselected patients in our cohort and in 36.9% of the total cohort of patients with malignant disease included after application of exclusion criteria and drop-outs a contact to SPC would have been triggered.

## Discussion

This study is to the authors’ knowledge the first validation of a screening procedure in German language identifying SPC needs of adult patients with advanced cancer and the first using filter questions as a pre-screening. We could show that an assessment of SPC needs in cancer care settings is feasible and the suggested tool presents a valid instrument for physicians to initiate a PC consultation.

The screening score for SPC needs of all screened patients was rated high by the physicians and the majority of patients were screened positively for SPC, probably caused by the filter criteria that limited the study population: a) an incurable and advanced cancer disease and b) that the physician would not be surprised if the admitted patient died within 1 year and by the fact that part of the study population was recruited from the PC ward. In particular the surprise question has been proposed for screening; however recent evidence from systematic reviews shows heterogeneous results regarding predictive value and accuracy. Downar et al. [26] found that “the surprise question performs poorly to modestly as a predictive tool for death”, however for cancer significantly better than for non-cancer diagnosis. White et al. [27] report a moderate accuracy, with a - compared to other diseases - higher sensitivity (79%) for cancer patients. Our study may add to the claim to determine whether the combination of the surprise question with other clinical indicators may improve the identification of patients with SPC needs. For clinical practice the use of filter questions ahead of a dedicated screening may be a feasible compromise between limited resources for screening on the one hand and adequate case-finding on the other hand. Additionally, it must be clear that with any tool also unscreened or negatively screened patients may develop needs, which should then lead to integration of specialized services.

Overall the screening tool showed satisfactory validity. Missing data in the IPOS lead to an exclusion of almost one third of the screened patients in analysis of convergent validity. As the IPOS sum score was used as an external criterion, we could only use those with less than half of all data missing. Imputing data in questionnaires with more than half of all data missing was not considered adequate. The high percentage of missing values in the IPOS might have been occurred due to the fact that some of the IPOS items query symptoms and psychosocial aspects, which may not be part of routine medical history and assessment in oncological settings (e.g. if the patient was in peace during the last 3 days). However, two-third of the screened patients could be included in the analysis, which showed a substantial correlation between the instruments as they both strive to measure important aspects in SPC. Concerning patient- and disease-related aspects the instrument is stable and independent and does not show significant differences in screening score between age, gender and primary diagnosis. While age and primary site showed no statistical differences, this reflects the small sample sizes of the subcategories. They show intuitive trends which would be significant in a large enough study designed to test these hypotheses. Effect of age is bimodal: Patients under 50 and over 80 have greater needs than those in the 50–80 age groups. As expected, patients already in contact to PC showed significantly higher screening scores than those without contact (9.27 vs. 5.44). It is noteworthy that almost half the patients screening positive were already known to SPC, acknowledging individual PC needs. This supports concurrent validity. In the study by Glare et al. [28] it was found that about two thirds of all admitted patients to a gastrointestinal oncology ward did screen positive but very few of them were previously known to the palliative care service. Furthermore patients that deceased during the study period had significantly higher values than those who didn’t (8.59 vs. 6.52), like it was demonstrated in a previous study by Glare/Chow [8] where the mean score was 5.0 when survival time was shorter than 6 months vs. a mean score of 3.3 when survival time was longer than 6 months. The overall higher sum scores in our study compared to Glare/Chow [8] may be due to the fact the population was preselected by the filter questions and about one third of the study population was recruited from the SPC ward.

Reliability of the screening tool showed moderate values in internal consistency, maybe caused by the heterogeneity and complexity of the evaluated phenomenon [23]. Deleting one of the items with low correlations would possibly lead to an impairment of content validity [19]. For inter-rater as well as test-retest reliability the instrument presents significantly substantial correlations between the screening scores of both estimations. Single items had a mostly good agreement. Four single items (‘one or more severe complications, often associated with a prognosis of < 12 months’; ‘patient/family concerns about course of disease and decision making’; ‘team needs assistance with complex decision making or determining goals of care’ and ‘prolonged length of stay (> average length of stay)’) showed poor agreement in inter-rater and test-retest estimations. Different levels of experiences of the physicians could constitute these differences as well as difficulties to rate these aspects in general. In test-retest changes of the aspects between first and second estimation are conceivable, which however couldn’t be captured by analyzing the SPI (e.g. if team needs support by PC consultation). Using a cut point of ≥5 showed needs of SPC consultation for 80.8% of the screened patients. Referring to all patients that were assessed this sums up to a proportion of 36.9%, which is comparable to a recent study of Glare/Chow using the same NCCN criteria (34% of assessed patients in needs) [8].

Calculations of positive and negative predictive values referred to the need for SPC treatment evident due to a preexistent contact to PC unit, inpatient or outpatient consultation team or IPOS estimations. Both definitions of patients in need are deficiently. Defining patients in need by judgment of a specialized palliative care professional (or patients own report) could help to determine the cut point more precisely.

Implementation of a screening tool for identifying SPC needs as presented here could improve clinical practice through timely and adequate SPC consultation in all oncological settings. However, issues of workload during daily routine for physicians have to be taken into account. Therefore we recommend the use of both filter questions to trigger the complete screening procedure.

### Study limitations

The study is based on four oncological settings within a larger comprehensive cancer center at one German university hospital. Therefore, the transferability of our results to other settings may be somewhat hampered. There will be needs in non-cancer patients as well, but those were not investigated during this study.

Above that, the study had a large proportion of non-responded and incomplete questionnaires (38.3%), although a study nurse accompanied the screening process by regular site visits and additional phone calls. This can be caused by the perceived length of the questionnaires (screening tool combined with IPOS), which may have be challenging to assess during daily routine. The staff-version of the IPOS was used for this study. We did not evaluate for how many patients it would have been possible to complete the patient version of the IPOS. Furthermore both questionnaires (screening tool and IPOS) capture aspects that may not be part of routine patient evaluation in oncological settings and could cause discomfort for physicians. In addition organizational challenges like short-term changes in attending physicians interfered with the recruitment process. Therefore it is necessary to examine implementation strategies, which assure minimal workload for screening in daily routine. Besides using the filter questions a review whether some of the screening items are already part of routine documentation may be helpful.

We do not have information on the patients with SPC needs we may have missed by the tool.

Furthermore we could only assign the deceased patient to the deceased/survival dichotomy, when the patient died during the study period and in the hospital setting. Information about patients that died after the study or in other settings was not yet available. That may have had an impact on the group sizes and their screening scores, respectively. This will be tested a planned follow-up study after 1 year after recruitment ended.

## Conclusions

This is the first validation of a phenomenological German screening tool based on the NCCN Palliative Care guidelines. It can be concluded that the screening tool satisfactory detects SPC needs of patients close to death that should lead to a PC consultation. A more adequate and timely case finding may be possible with our suggested combination of two filter questions and a dedicated questionnaire.

## Data Availability

All data is presented in the manuscript. The datasets analyzed during this study are available from the corresponding author on reasonable request.

## Abbreviations

IPOS: The Integrated Palliative Outcome Scale
PC: Palliative Care
SPC: Specialized Palliative Care
